# Suicide: The pandemic inside the pandemic

**DOI:** 10.1192/j.eurpsy.2021.728

**Published:** 2021-08-13

**Authors:** R. André, C. Sereijo, M. Abreu

**Affiliations:** 1 Psiquiatria, Centro Hospitalar Universitario Lisboa Norte, oeiras, Portugal; 2 Psiquiatria, Centro Hospitalar Universitario Lisboa Norte, Lisboa, Portugal

**Keywords:** COVID-19, Suicide, SARS-Cov2

## Abstract

**Introduction:**

Covid-19 was declared a pandemic by the WHO on March 11th and efforts have been made to minimize the impact that this new disease can produce. The mental health effects of this pandemic can be severe considering that each year close to 800.000 people die by suicide. This pandemic could increase those numbers, although this is not inevitable.

**Objectives:**

This work reviewed the current available data on possible causes for a suicide rate increase and to try to understand if suicide is already increasing worldwide.

**Methods:**

Non-systematic review of the literature with selection of scientific articles published in the past 6 months; by searching Pubmed and Medscape databases using the combination of MeSH descriptors. The following MeSH terms were used: Covid-19; suicide; SARS-Cov2; pandemic.

**Results:**

Multiple factors can account for an increase in suicide rates such as isolation with a sense of decreased belongingness and increased burdensomeness. A synergy with known suicide precipitants can also occur such as domestic violence, intra-familiar conflicts, alcohol consumption and access to means. Media communication can represent a danger with constant reports about the crisis. And lastly the loss of employment and financial stressors can produce an important impact.

**Conclusions:**

In conclusion, Covid-19 will produce an important impact in many spheres of society, one of which will be mental health. If at the start of this crisis a widespread sense of solidarity was present with the maintenance of precipitant factors for suicide we expect to see an increase in suicide rates.

